# Ectopic Gestation, with Report of Cases

**Published:** 1896-02

**Authors:** F. S. Love

**Affiliations:** San Antonio


For Texas Medical Journal.

### ECTOPIC GESTATION, WITH REPORT OF CASES.

BY F. S. LOVE, M. D., SAN ANTONIO.

Read before the West Texas Medical Association.

IN TAKING up the subject of extra-uterine pregnancy, or ectopic gestation, I am fully aware of the fact that much is said and written on the subject to-day. It is not my purpose to challenge any one's views, but to state a few facts and then report a case or two to sustain my statements. By the term extra-uterine pregnancy we mean any pregnancy that exists outside of the uterus; thus an interstitial pregnancy is not, properly speaking, extra-uterine, while all pregnancies existing outside of uterine cavity are ectopic. So we are forced to the conclusion that all ectopic pregnancies are not extra-uterine. Yet all extra-uterine pregnancies are necessarily ectopic; therefore, why not use the term ectopic in all cases? Ectopic pregnancies were considered very rare occurrences until of recent years; now we find it no rare thing to meet with them. Formad, of Philadelphia, claims to have found, in a series of 3500 general autopsies, thirty-five were ectopic gestations.

The first reported case of ectopic gestation was reported about the middle of the eleventh century by Albucasis, then we heard very little of this interesting trouble until 1883, when Tait reported his first successful operation for the removal of a ruptured tubal gestation. Since his report of his case, the journals have been flooded with reports of cases and articles on the subject.

We meet with several varieties of ectopic gestation—tubal, tubo-ovarian, tubo-abdominal, tubo-uterine or interstitial. However, the most common form met with is the tubal. Some surgeons claim the ovarian form does not exist, yet other good authorities report cases to prove their statements. Zedel reports a case where the enlarged ovary contained the fœtus, the microscope showing the flat and round bones and the membrane of a fœtus. Where the existing conditions are favorable, I do not see why we should not have the ovarian form as well as the tubal. Of the abdominal form there are equally as many different opinions. Some claim the abdominal pregnancies exist free as such from the start; but the weight of the argument is in favor of their being the result of tubal abortion or rupture. It is fallacy to suppose an ovum could exist in the peritoneal fluid long enough to meet with the spermatozoon, conception take place and develop. We know the peritoneum is able to absorb much more resisting masses, and before the circulation of the ovum could be established death would be the result, and absorption follow. That abdominal pregnancies exist as the result of tubal abortions or rupture, is a well established fact. Yet, in most of the cases the fœtus dies from the hemorrhages, although some few escape death, even when the rupture is into the abdominal cavity. But when the rupture is into the folds of the broad ligament, the pregnancy may go to full term. The interstitial variety is not so common as the tubal, and the rupture in these cases is more apt to prove fatal. That ectopic gestations exist is a settled fact. But why? That is not so easily settled. I think most of the best authorities favor the view of some change in the tube. Any change that would lessen the lumen of the tube, such as an inflammatory process of whatever cause, displacement of tube, torsion, or any change that will enlarge the vessel, such as dilatation, casting off the ciliary epithelium, to chronic salpingitis, or any cause that will destroy or weaken the ciliary movement, and peristalsis, which carries the ovum to the uterus. Yet these changes do not interfere with the passage of the spermatozoon from the uterus to the tube. Yet the tube does not have to be diseased to insure a case of ectopic gestation, for Tait has reported several cases where the microscope failed to show any deviation from the normal tube. Lusk reports a very interesting case where the conception took place in the right tube which was not diseased, and the ovum coming from the left ovary, as the fresh ruptured follicle was found in the left ovary. To prove that the ovum could migrate externally from one ovary to the other tube and hence into the uterus, Leopold tied the right tube, and removed the entire left ovary from two rabbits, and uterine pregnancy took place in both cases. The internal migration of the ovum is still a much disputed fact, as we know the wave-like movement of the ciliated epithelium and peristalsis of the tube, which carries the ovum from the ovary to the tube, will not allow its passage from the uterus into the tube. Again, we find that failure of development of tube, or the one-horned uterus, may be predisposed to this mishap, or the presence of a mucous polypus within the tube may act as a factor obstructing the way, allowing the spermatozoon to reach the ovum, but not permitting the ovum to pass, owing to its increased size after fecundation. There seems to be a disposition for these mishaps to recur in the same patient. Coe has reported a very interesting case in which tubal pregnancy occurred the second time in the same tube.

Statistics show a few cases of ectopic pregnancies existing with uterine pregnancy, but they are very rare, if ever found at all.

When the fecundated ovum becomes lodged in the fallopian tube, the tube becomes thickened at the point of contact with the ovum, caused by the increased circulation. The muscular coat of tube is strengthened and develops slightly; as the ovum develops, the fibriated end of tube retracts, and the peritoneal and muscular coats extend over it and seals the opening. This closing process is completed about the eighth or tenth week. So all tubal abortions must take place early in pregnancy. As the ovum grows the increased pressure in the tube and the stretching of its small wall causes it to become thinner as the gestation advances, and finally rupture is the result, which occurs in about eight or ten weeks; but if the pregnancy exists in the uterine end of tube, it may rupture even earlier, as the isthmus is more friable and will not admit of so much distension as the distal end of tube. The rupture may be caused by a misstep, a fall, a blow, and some one has reported a case where the rupture was traced directly to sexual intercourse. Then the changes within the tube are often the cause of rupture, as rupture of a small vessel, causing hemorrhage, which so distends the tube that rupture follows. The ovum undergoes the same changes in tubal pregnancy as in uterine, having the same membranes, only not so thoroughly developed.

As stated above, nearly all of the ruptures mean death to the fœtus, and many are fatal to the patient. In all cases which are not fatal, we find the membranes have formed a plug, this closing the opening and for a time saving the patient's life. The membranes have a tendency to contract on themselves, thus forming a cup-like mass, which has a tendency to check the hemorrhage. The peritoneum is excited at once by this effusion of blood, and throws out its plastic material which wall off this mass from the peritoneal cavity. If this first hemorrhage is not fatal to fœtus or mother, the placenta may repair its torn surface by extending its attachments to the adjoining vicera and fœtal life may continue. But as a rule recurrent hemorrhage takes place which proves fatal to fœtus and often to mother. Again, we may have a secondary rupture; that is, when the first rupture occurs into the folds of the broad ligament, making it extra peritoneal, the broad ligament may become so distended either from the growth of the fœtus or from hemorrhage, that it will rupture into the peritoneal cavity proper. These secondary ruptures are always fatal, as they occur so suddenly and without warning, that the patient is in a state of callapse in a few moments.

In tubal pregnancy the formation of the uterine-decidua is the same as in normal pregnancy, with the exception of a less marked division of its three layers. When a patient presents herself before her physician, suffering from a mishap of this nature, she believes herself to be pregnant, yet the pregnancy is advancing satisfactorily. She first notices some change in her menses, scanty or stopped altogether—these symptoms give her little or no concern; in factr the early ectopic gestation does not differ materially from the normal; but later she is startled by sharp lancinating pains in one side of the abdomen, which is at times so severe that she grows faint and may lose consciousness. These distressing symptoms are usually followed by a return of uterine hemorrhage and the discharge of the uterine decidua; this discharge may continue for two or even three weeks, the patient thinking she has had a miscarriage; then peritonitis follows; it may subside and the patient flatter herself her troubles are ended, when she again falls a victim to the same suffering, and often the suffering is more acute. You will often find a history of sterility of long standing, but not always. To sum up the symptoms we find the following: amenorrhœa, symptoms of pregnancy, sudden sharp pain with or without syncope, history of sterility. If examined before a rupture, a case of ectopic gestation is not easily made out; we find the tube an enlarged, sausage-shaped mass, more boggy than a hydro or pyosal pinx; the uterus is usually enlarged and may or may not be displaced by the tumor dragging or pushing it out of place. The cervix is soft, velvety to the touch, and the internal os is often plugged with mucus. Yet we sometimes find that the uterus and cervic do not show any change. With these symptoms well established, in connection with the patient's history, help us to diagnose an ectopic gestation. When rupture has taken place, which is often the case before a patient presents herself for treatment, we either find a hematoma or a hematomacele existing. In case of hematoma, it is usually formed in the broad ligament. Here we may err in our diagnosis, unless we get a clear history of the case, which, in some cases, is withheld. As hematoma may be caused by rupture of veins during a congested condition of these parts, and lead us astray. Again, in hematocele the free blood will gravitate into the pouch of Douglass, and may fill the pelvic cavity, pushing the uterus out of its normal position. The blood coagulates in time and is roofed off by peritoneal exudate, forming, as absorption takes place, very strong adhesions with the abdominal viscera. Nearly, if not all hemotoceles, are the results of tubal abortions or ruptures, and their existence should make us suspicious of an ectopic gestation, and the diagnosis is to be given guardedly until a full history is obtained. There are many conditions of diseased ovaries, and tubes, and even a fibro-myoma may so nearly resemble this mishap as to lead us into gross error. Often the history is misleading, for in some cases there is no deviation from the normal and in some cases the symptoms of pregnancy are withheld. Now, suppose we have a case of ectopic gestation. What will we do? This question is more easily asked than answered. Provided we see the case before abortion or rupture, we are told by experience of good men, to inject the gestation sac with atropine or morphine, or use the Faradic current, with the view of killing the fœtus. Granting we are successful in bringing about this great end, the death of this offending fœtus, is our patient safe? Have we not the dead fœtus now to contend with, instead of the living one? Have we made any headway toward the desired end? Are we not playing a game of chance by using these means? Are the dangers of rupture ended with the life of a fœtus? Lessened, we admit, but not ended. So long as this fœtus remains, living or dead, the danger of internal hemorrhage is to be dreaded. Electricity, we know, will kill the fœtus, and that it hastens abortion, we grant. Yet it takes time, the all-powerful agent in these cases. Even the most ardent advocates of this agent says it is not safe after the third or fourth month. Can we expect these thickened organs, bound together as they are with inflammatory exudate, to absorb this mass? I think not With the present knowledge of abdominal surgery, and the almost perfect technique of today, it is far better to do the radical operation for the removal of this mass, which is so prone to cause trouble later on. The expectant plan is tardy justice to our patient. The surest way to get our patient out of danger, and I might add, the quickest, is to remove this gestation sac as if it was a foreign body. When left, after death of the fœtus, in close proximity to the rectum, this mass is more than likely to suppurate, and we all know the unpleasant results of these ugly pelvic abscesses. Again, we do not say that where the abortion or early rupture occurs that the abdominal viscera is not able to absorb a large portion of this mass, and in many cases, no doubt, it does this work and a good recovery follows. But, if the hemorrhage is copious, it is best to open the abdomen, and, with the eyes on the field, all vessels can be taken up and secured, for who can say when this hemorrhage will return, or when the case may end fatally? Although the best surgeons of the age claim, it is bad practice to leave it to nature, be the hemorrhage ever so slight,—and I make the statement again,—it is injustice to ourselves and our patients, to tamper with life by delaying to give them the benefit of an abdominal section. There is much to be said on the subject, but I shall not tax your patience further, but proceed to the report of a few cases that I think will interest you more than the rehearsal of these well known facts.

The first case that I will call your attention to is of a lady 26 years old, married five years, no children, no miscarriages, family history good, had had all the diseases of childhood and made good recoveries, menstrated first at 14 years, after it was established, always regular and painless. In August noticed a change in menses, scanty and watery. None appeared in September, and thought herself pregnant. Late in September she felt sharp knife-like pains in left side of abdomen. This continued for two or three weeks, when she fell in a faint. Some days after the uterine hermorrhage made its appearance. Thinking she had had a mascarriage, was up and around, but still the pain kept up, and she suffered from pressure symptoms. Late in December (five months after the trouble), she came to the hos pital for treatment.

Examination disclosed a mass in the pouch of Douglas; taking the history, which was clear, with the examination, ectopic gestation. Accordingly the belly wall was opened, found the placenta after much trouble. All the abdominal vicera was mattered together by strong adhesions. The fœtus could not be found. The placenta was soft and partially broken down. It was removed, belly flushed out, and to check the oozing hemorrhage, the pouch of Douglas was packed with a Mikilicy drain, containing 1½ yards of iodoform gauze. The wound was then closed around the packing and dressed. Twelve hours later the packing was changed, and removed entirely about the fourth day. Patient made a good recovery, and was dismissed in about six weeks.

SECOND CASE: Mrs. J., age 35; widow, history misleading; complained of weight in the pelvis and soreness for some months; said menses regular, not changed in any way. Diagnosis, pelvic abscess; proceeded to open per vagina; to our surprise a dark blood clot rolled out through the incision. This at once gave a suspicious look to things, so we opened the belly wall and found a large amouut of congulated blood, and a ruptured tube containing the fœtal membranes. We removed the tube, but it did not contain the fœtus; then flushing the cavity out found the fœtus, which was about 1½ inches in length. It was free in abdominal cavity. There being no bleeding surfaces we closed the wound with silk-worm gut, interrupted sutures with drainage per vagina, using iodoform gauze. Patient left hospital in five weeks, and in seven weeks was able to be around.

THIRD CASE: Mrs. M., age 42; mother of six children, had two miscarriages. Being a very poor woman, did not call in medical help, thinking she was pregnant and time would cure her ills. From the history we are forced to conclude the rupture must have taken place about the third month, for she said she thought for a time that she miscarried about that time. She suffered on for months without medical aid; died very suddenly. At post mortem examination the belly was found full of blood clots and a fœtus of full term or nearly so, was found in the folds of the broad ligament on the right side; child had a fine head of hair, nails perfect, and was as finely developed a child as I ever saw. This case shows that the fœtus may live and develop after abortion or rupture, and death to mother caused from secondary rupture. If she had called in aid sooner, and the trouble been recognized, she, mother and child, might have been saved.

FOURTH CASE: Mrs. B., age 27; two children, no miscarriage, last child five years old; health perfect, menses normal. Had felt sickening pain in side for a day or so; was out walking when she slipped, only remembered severe pain; unconscious. When we saw her she was suffering from severe shock and internal hemorrhage. We made a hasty section, and found the right tube ruptured and still bleeding; secured and removed it at once, found fœtus in the blood clots deep in pelvis; flushed abdominal cavity out with hot sterilized water, and putting about a gallon of hot sterilized salt solution in the belly to overcome shock, closed the wound and dressed as in ordinary case of section; patient rallied in about six hours, but was very weak. Made a slow, tedious recovery, and was dismissed in about ten weeks.

I think these four cases sufficient to show the different ways this mishap may effect a patient, and to put us on the lookout for ectopic gestation.

